# Two Memoirs Read before the Royal Academy of Sciences, at Paris, on the Inhalation of Chlorine in Pulmonary Consumption

**Published:** 1831-01-01

**Authors:** 


**TWO MEMOIRS READ BEFORE L'ACADEMIE ROYALE DES SCIENCES, AT PARIS, ON THE SUCCESSFUL INHALATION OF DILUTED CHLORINE IN THE EARLY STAGES OF PULMONARY CONSUMPTION, &c. Translated from the French of *M. Gannal*, by *W. H. Potter*, M.R.I. Operative Chemist. Octavo, London, 1830.**

THE sanguine expectations which were elicited by the theories of Lavoisier, and the ardent imaginations of Beddoes and others, respecting the efficacy of gases inhaled for the cure of breaches of structure in the lungs, have long been dissipated before the clearer lights which have been shed on pulmonary consumption by pathological anatomy. Oxygen gas was to have done as much in former times, as St. John Long's liniment and insufflations are now said to do—and the bubble generally lasts long enough to run the philanthrophic enthusiast into an ocean of absurdities, and put abundance of money into the pocket of the charlatan. There is an hallucination attendant on phthisis, which will ever produce a rich harvest for unprincipled quacks and impudent pretenders. The phthisical patient is so confident of recovery, that every secret medicine administered to him is lauded as producing miraculous effects—and these effects are asserted with the last gulp of air which he draws into a disorganized mass of lungs! To open the eyes of the community, then, towards the fallacies of pretended specifics, and the mendacious assertions of quacks is utterly impossible, and we have long since seen the impracticability of the attempt. The community *will* be deceived—indeed, they almost *wish* to be deceived—and as the exuberance of population has ever been represented as a destructive evil, in all ages and in all countries, it would be vain—perhaps it would be impious, to stem an impulse or instinct, which appears to be implanted in human nature for some wise purpose by the omniscient Ruler of the Universe.

The medical philosopher, however, has nothing to do with this part of political arithmetic. The man who tells you that exuberance of population is an evil—and who points out to you the various checks to this exuberance, consults you, at the same moment, on the best means of increasing the number of his family, of preserving their health—and of prolonging his own existence! It is to this individual feeling that the medical man has to direct his attention—and not to the abstract doctrine of population.

But to return. Notwithstanding the failure of inhaling oxygen and other gases—the fumes of tar, and other medicinal substances, there is still a fond hankering after the direct application of remedies to the ulcerated cavities of the lungs—an application which can only be made through the medium of the air we breathe.

"But now that chemistry, which at first sight appeared to have invaded the province of medicine, by furnishing some certain remedies against the diseases to which we are exposed, is at last relieved from the discredit which has unjustly been heaped upon its application to the healing art, it remains to discover, if amongst the substances revealed to us, by its means, there is not one which may produce the favourable effects of oxygen, without lying open to the same objections.

That such a substance exists is most evident, and this is CHLORINE, first studied by Guyton Morveau, who pointed out many of its applications. We are now well acquainted with its extensive utility as a disinfecting agent, and with its energetic action on animal matter generally. Recent facts, published by physicians of first-rate eminence, have shewn that it powerfully modifies organic action, cleanses and neutralizes the matter of chronic ulcers, assigns limits to mortification, suppresses long-accustomed mucous evacuations, and may be given internally in scorbutic cases with advantage, as also in putrid fevers and other similar diseases.

Most of these facts have been pointed out in the works of Guyton Morveau, Hallé, and Fourcroy. But thanks to the exertions of an esteemed pharmacien, thanks to that zeal for the general good which leads men in these days to unite their efforts to procure the triumph of all that is useful, the applications of Chlorine, for instance, combined in excess with the metallic oxides, to which it adheres but slightly, have been rendered general, and crowned with abundant success.

Four years of experience have contributed more to setting off the advantages of this new discovery, than the twenty years which followed the fine researches of Guyton Morveau. We may then conclude from the mass of knowledge now acquired respecting Chlorine, that it not only destroys putrid animal exhalations, and is on this account the most powerful disinfectant we possess, but that it exerts a salutary influence, still more strongly marked, upon living beings themselves, and powerfully modifies the organic actions by which they are distinguished." 14.

It now remains to inquire whether this same chlorine may be administered with advantage in cases of pulmonary consumption. Analogy, says the author, would lead us to conclude that, since the remedy has been found useful against copious mucous discharges from the vagina—ill-conditioned ulcers, and gangrene, it should produce similar effects in the ulcers and their secretions constituting phthisis. The probability of this reasoning, M. Gannal thinks, was converted into certainty, by the fact of his observing, in 1817, that the printed calico-manufacturers of St. Denis, who happened to be affected with phthisis, quickly recovered their health while exposed to the exhalations of the chlorine disengaged in the various processes. Laennec tried the remedy in La Charité; but either did not find it efficient, or did not follow up the plan with sufficient energy. Since that period the author avers that he has had several opportunities of witnessing the good effects of chlorine in consumption and pulmonary complaints. The following results are given by the author to shew the success of the remedy.

" First of all I must point out the manner in which I have been accustomed to administer the gas. In the fumigations recommended by Guyton Morveau, the Chlorine produced in a state nearly dry and in great quantity, often mixed with muriatic acid gas, and also contaminated with small quantities of sulphuric acid carried over during the operation, produced violent irritation in the pulmonary passages; an intense heat; a feeling of oppression; and soon after, a sharp fit of coughing were the effects of its inhalation. Thus it was found necessary before the disinfecting process was commenced, to clear the rooms, and when the Chlorine was disengaged in occupied apartments, the apparatus was obliged to be moved away to some distance from the sick, who often, notwithstanding the greatest precautions, were much inconvenienced, and coughed violently.

This mode of procedure could not therefore be applicable to phthisical patients.

Guyton Morveau had proposed to substitute for the bottles of acetic acid, and other substances of but little efficacy, bottles of chloruretted oxide; but even this means, which has since proved of such general application, was not itself free from objections.

I shall not undertake to determine whether the Chlorine evolved from Chloruretted oxides, differ in a chemical point of view from that which in a perfectly pure state is dissolved in water. However, I am inclined to believe that such is the case; the smell of this Chlorine is manifestly not the same as of that procured in the ordinary method, by the mutual action of hydrochloric (muriatic) acid and oxide of manganese. Though I will not at this time venture to speak positively on this point, I think that the Chlorine disengaged from the chloruretted oxides (potass, soda, and lime) is contaminated with some particles of a foreign nature, which affect its purity when it is immediately applied to the delicate organs of respiration. This statement is rendered more probable by considering what follows: I caused some of my patients to respire Chlorine evolved from a chloruretted oxide, but at the third fumigation they experienced a lively sensation of warmth in the chest, constriction of the throat, thirst, and all the signs appertaining to a powerful stimulus, which made me quickly desist. Chlorine then, as produced from its combination with oxides, is not of sufficient purity to warrant our applying it to the delicate and already irritated organs of the phthisical patient.

To remedy this inconvenience, I make use of a solution of the pure gas in distilled water. I take a three-necked bottle, the first opening receives a straight tube, the extremity being plunged into about four ounces of water, the second opening has a tube, which leaving the top of the bottle is bent at right angles, and terminates in a flattened embouchure, the third is furnished with a glass stopper;* it is by this last opening that the water is changed, and the gas introduced. The water in the bottle at the time of fumigation should be at the temperature of about 32° of the centigrade thermometer equal to 89° 6′ of Fahrenheit's scale; a certain quantity of liquid Chlorine is then added; and by gently shaking the bottle a portion of the gas is disengaged, which may be breathed by applying the mouth to the extremity of the bent tube. As the air is gradually withdrawn from the bottle, a fresh quantity is supplied from the atmosphere by the straight tube, bubbles up through the weak Chlorine solution charged with the gas. The fumigation may be continued for the space of four or six minutes, after which the disengagement of gas ceases.

It is of the utmost importance, that we proceed with the greatest caution, being guided by the consideration of the energetic nature of the means employed, as also of the delicate fabric of the organ concerned. In my practice I commence with ten drops of the liquid Chlorine to two volumes: if the patient can well bear this dose, and according to the susceptibility of his lungs; I raise it gradually to 12, 15, 20, 30, 50, 60, 72 at a time. However, there are scarcely two persons who can bear exactly the same doses; we must therefore, as it were, carefully explore the state of the organ to be acted upon, and from thence deduce the proper quantity.

The same reasoning holds good as regards the *number* of fumigations, during the twenty-four hours. They must be regulated by the effect produced, and the sensibility of the parts, generally the number may be from six to eight. It appears evident according to the above process, that the Chlorine inhaled cannot enter the lungs unless impregnated with a number of aqueous particles; from which circumstance it is much less irritating than in the dry state; and lastly, not being commixed with any foreign matter, its action confined to itself is not complicated with any superadded irritation." 22.

---

* "The apparatus fitted up complete, as recommended by M. Gannal, as also the solution of Chlorine gas, in a perfectly pure state, may be obtained at the Chemical Laboratory, No. 11, Old Compton Street."

The apparatus for the exhibition of this gas is simple and easily obtained. It is portable, and being made of glass, the chlorine cannot become contaminated by metallic molecules. It has happened to this as to many other new remedies—that patients irrecoverably gone in phthisis have had recourse to the measure without benefit. We cannot do better than introduce some of the cases which the author has adduced as verifying the salutary effects of the remedy under consideration.

"The first is that of M. L * * * *, of Gentilly, this man was 40 years of age, of a lymphatic and bilious temperament, and had been troubled a long time with pectoral disease when he applied to me. I sent him to Dr. Laennec (nephew of the professor); this physician after examination found that the chest under the right clavicle had a more dull sound than under the left; respiration sufficiently free throughout the right side, was *caverneuse* under the arm-pit and clavicle, and accompanied with a sort of moist rattle; in the left portion, respiration was natural, only accompanied here and there by a slight wheezing.

From these symptoms M. Laennec announced the existence of a tubercle on the top of the right lung, and 'I dare even affirm,' he adds, 'that the whole of this lung is more or less covered with tubercles of various magnitudes, indicated by the peculiar and variable sound attending respiration, as also by the crackling rattle. I think,' says M. Laennec, in conclusion, 'that this case is one in which the Chlorine fumigations may be tried, but with discretion, however, on account of the disposition to hæmoptysis, and to pleuritic inflammation.'

This man who had been ill three years, commenced on the 18th October, 1827, to use the fumigations, the dose being ten drops, eight times a day, from the 18th to the 23rd; respiration became less obstructed, the sense of oppression was much diminished, the expectorated matter from being purulent assumed a mucous appearance, the diarrhœa ceased, appetite returned, and the digestive organs again performed their healthy functions, and he passed more tranquil nights.

On the 23rd, however, from eating herrings, symptoms of indigestion were produced, the expectoration was tinged with blood, but notwithstanding this, the Chlorine was still continued, and the patient gradually progressed. No inconvenience was felt from breathing the gas, the patient seemed extremely sensible of the least atmospheric change, colicy pains were experienced on the 17th of December, but were relieved by emollient applications, the appetite continued good, digestion went on well, the patient had one stool daily, his nights were undisturbed, the nocturnal perspirations were not so frequent, the expectorated matter purulent in the morning was mucous during the day. The sense of oppression is almost gone, and the cough is much less frequent. On the 23d of December, the pulse was 62. The season was much against the successful exhibition of the remedy, and if the man is not cured, we cannot refuse to admit that the Chlorine on the one hand, never incommoded him, and on the other, produced such alleviation that without doubt the patient's life was evidently prolonged.

The second case I shall mention is that of M. D——; I cannot better describe this than by copying the report of his physician, Dr. Honlet, to one of his friends who desired the particulars. 'Consulted early in the month of September, by M. D—, I declared him a consumptive patient, so far advanced in the disease, as to judge him incurable. On the 8th of the following October, by the advice of somebody whose name I do not recollect, Chlorine fumigations were proposed, as a means likely to effect a cure; and, as you may imagine, most cheerfully acceded to on my part; so much the more as this mode of treatment was totally unknown to me. As a farther proof of its beneficial action, I declare candidly that not only was this very evident to myself, but also to each of the other physicians (M M. Cretin and Guersent) who also saw this patient.

'The fever and the coughing fits yielded to the remedy instantly: the digestive organs experienced a freedom of function hitherto unknown: the cough was now less frequent; and the sputas from a purulent character became changed to muco-purulent, and lastly more decidedly mucous. The fumigations were repeated four, five, and six times a day, three or four minutes at a time, and these which were more or less charged with Chlorine, sustained our hopes for some time. The patient taking advantage of his partial restoration, ventured to walk from the Faubourg St. Martin to la Place Royale; but at his return his imprudence cost him much, he was seized with a fit of coughing, blood was expectorated, attended by a febrile paroxysm, from all of which it was evident that the mucous surfaces of the bronchiæ and stomachic viscera becoming the seat of disease, extended their morbid action to the neighbouring lungs.

'All hope was now at an end, and the patient given over. The fumigations were, however, still persisted in at the wish of the patient, for he was in the habit of saying, and repeated the same to-day, that 'they expanded his breast and stomach, and procured him some internal delightful sensations.' (These expressions were copied in a letter of the 19th December, 1827.)

The third case I shall mention is M. Le Compte de * * *, who had been ill for about two years, he had taken large quantities of pectoral medicines, a blister had been applied to his arm when he left Belgium to return to Paris, according to the advice of his physician. His cough was troublesome, obstinate, always accompanied by purulent expectorations; he slept only at short intervals; a dull sound was heard at the upper part of the left lung. M. D., of a dry and warm temperament, by the advice of several physicians, whom he had consulted, continued the plan of treatment already pointed out, and was recommended by them to pass the winter at Nice, in Italy, or in some other southern country.

When I was called to him I commenced the Chlorine fumigations on the 21st October, 1827. Their first effects were to diminish the frequency of the cough, and the quantity of expectorated matter. The appetite improved, colour returned to the cheeks, the skin remaining dry and hard, baths were ordered, and Dr. Begin recommended a mild regimen. The health of the patient has been improving since the 1st of November, and excepting certain unfavorable changes produced by atmospheric variation, things have worn a promising aspect, and at present there is a well grounded hope of a perfect cure. The peculiar sound in the left lung has ceased, the appetite is good, the nights tranquil, the usual strength recovered, and every thing shows fair for a speedy restoration."* 31.

M. Gannal had commenced experiments in various hospitals, but the results were not sufficiently matured when his work went to press. He avers that every one of his patients "received some relief"—"every one has had his respiration rendered more free—his expectoration diminished—the sense of oppression lessened." No increase of heat, fever, or hæmoptysis has occurred from the remedy. All these facts, he thinks, indicate a diminution of pulmonary irritation, and an increase of vital energy,

In a second memoir read before the Academy of Sciences, M. Gannal has detailed some more cases, a few successful, but most of them the reverse. It is to be acknowledged indeed, that the subjects for experiment were of a very unpromising kind, and therefore the chlorine only failed where every other remedy would have shared the same fate. We confess, however,

---

* The first and second case died soon after the memoir was concluded. The third, M. Le Comte de * * * * recovered entirely.

that we do not entertain any very sanguine hopes from inhalation of chlorine, or of any other medicinal substance, in real tubercular diseases; though we would not discourage fair trials of any remedy in such a deplorable malady.

